# LINC00312 represses proliferation and metastasis of colorectal cancer cells by regulation of miR‐21

**DOI:** 10.1111/jcmm.13830

**Published:** 2018-08-22

**Authors:** Gang Li, Changming Wang, Yongbing Wang, Bin Xu, Wenzhong Zhang

**Affiliations:** ^1^ Department of General Surgery Shanghai Pudong New Area People Hospital affiliated to Shanghai University of Medicine & Health Sciences Shanghai China; ^2^ Department of Gastrointestinal Surgery Changzheng Hospital Second Military Medical University Shanghai China

**Keywords:** colorectal cancer, LINC00312, metastasis, miR‐21, PTEN

## Abstract

Long non‐coding RNAs (lncRNAs) have emerged as important regulators of cancer, including colorectal cancer (CRC). The exact expression pattern of long intergenic noncoding RNA 00312 (LINC00312) in CRC and its mechanisms of action have not been reported. Here, we found that LINC00312 is underexpressed in CRC tissues and cell lines. Functional experiments suggested that LINC00312 suppresses growth, migration and invasion of CRC cells in vitro and attenuates tumour proliferation and metastasis in vivo. Mechanistically, LINC00312 was found to regulate the malignancy of CRC cells by binding to miR‐21 and by functioning as a tumour suppressor targeting PTEN. Overexpression of miR‐21 or knockdown of PTEN attenuated the LINC00312‐mediated inhibition of CRC cell proliferation and invasion. Taken together, our results elucidate the role of the LINC00312–miR‐21–PTEN axis in CRC cell proliferation and tumour progression and may lead to new lncRNA‐based diagnostics or therapeutics for CRC.

## 1 INTRODUCTION

Colorectal cancer (CRC) is ranked as the third most prevalent cancer and occurs in both men and women, with ~700 000 deaths per year.^1^, ^2^ Therapeutic methods including surgical resection, chemotherapy and radiotherapy have been widely used but poorly improved as CRC treatments.^3^ Therefore, exploring the molecular mechanisms such as inactivation of tumour suppressor genes might provide significant insights into adequate improvement of diagnosis and management of CRC.

By means of next‐generation sequencing, long non‐coding RNAs (lncRNAs) longer than 200 nt have been identified. Accumulating evidence regarding lncRNAs has shown their possible roles in various cancers, including CRC.^4^, ^5^, ^6^ The latest study revealed that lncRNA CRNDE may promote CRC cell proliferation and chemoresistance by modulating miR‐181a‐5p and the Wnt/β‐catenin signalling pathway.^7^ In addition, a novel lincRNA termed “UCC” was found to be highly expressed in human CRC tissues and cell lines and to facilitate CRC progression by acting as a sponge for miR‐143.^8^ The expression of lnc‐sox5 is significantly higher in CRC and regulates CRC tumourigenesis by unbalancing the tumour microenvironment.^9^ A long intergenic non‐coding RNA called LINC00312, encoded in chromosomal region 3p25.3, has been reported to be a novel tumour suppressor gene in several cancers,^10^, ^11^, ^12^ but its role and mechanism of action in CRC development remain to be elucidated.

In this study, we observed that LINC00312 is underexpressed both in CRC tissues and in cell lines. LINC00312 overexpression suppressed growth, migration and invasion of CRC cells in vitro and attenuated tumour proliferation and metastasis in vivo. Furthermore, we found that LINC00312 inhibits the progression of CRC by modulating miR‐21–PTEN signalling. Our work provides evidence of a cross‐talk between LINC00312 and miR‐21 and suggests that LINC00312 may serve as a possible new target for the treatment of CRC.

## 2 MATERIALS AND METHODS

### 2.1 Collection of CRC samples

Twenty‐two CRC specimens and their matched adjacent normal tissues were collected at the time of surgical resection at Shanghai Pudong New Area People Hospital affiliated to Shanghai University of Medicine & Health Sciences (Shanghai, China) in 2016. All the patients have not received preoperative chemotherapy or radiotherapy. All the tissues were immediately snap‐frozen in liquid nitrogen after resection and stored at −80°C. The study protocol was approved by our institutional review board, and informed consent was obtained from all the patients.

### 2.2 Cell culture

Human CRC cell lines SW480, SW620, LoVo and HT29 were obtained from the Institute of Biochemistry and Cell Biology of the Chinese Academy of Sciences (Shanghai, China) and were cultured in Dulbecco's modified Eagle's medium (DMEM; GIBCO, Grand Island, NY) supplemented with 10% of foetal bovine serum (FBS; HyClone, Logan, UT). The normal colon epithelial cell line NCM460 was grown in DMEM:F12 (GIBCO) supplemented with 10% of FBS. The cells were maintained in a humidified incubator at 37°C and 5% CO_2_.

### 2.3 Transfection and lentivirus transduction

An miR‐21 mimic, a miR‐21 inhibitor, PTEN siRNA and their respective negative control (NC) oligos were purchased from Genepharma Co., Ltd. (Shanghai, China). Oligonucleotide transfection was performed with Lipofectamine 2000 reagent (Invitrogen, Carlsbad, CA, USA). The complementary DNA encoding LINC00312 was cloned into the pCDH‐CMV‐MCS‐EF1‐coGFP construct (System Biosciences, CA, USA) to generate the pCDH‐CMV‐LINC00312 expression vector. The production and purification of lentivirus particles were conducted as described by Zheng and colleagues.^13^ The packaged lentivirus particles were named Lv‐LINC00312. The empty lentiviral vector Lv‐control served as a control. Cells were infected with recombinant lentivirus‐transducing units plus 5 mg/mL Polybrene (Sigma, St. Louis, MO, USA).

### 2.4 RNA extraction and quantitative reverse‐transcription PCR (qRT‐PCR)

Total RNA was isolated from tissues and cell lines with the TRIzol reagent (Invitrogen, Carlsbad, CA, USA). RNA was reverse‐transcribed to cDNA by means of the PrimeScript RT Reagent Kit (TaKaRa, Dalian, China). SYBR Premix Ex Taq (TaKaRa) was used to detect LINC00312 and miR‐21 expression. PCR was carried out at least in triplicate, and the results were analysed on an ABI 7500 Fast Real‐Time PCR System (Applied Biosystems, Foster City, CA). LINC00312 and miR‐21 expression levels were normalized to the expression of *GAPDH* and *U6*, respectively. The 2^−ΔΔCT^ method was applied to measure the relative expression of mRNA.^14^

### 2.5 Western blotting

Cells were lysed with RIPA extraction reagent (Thermo Fisher Scientific, MA, USA) to obtain total protein. Total protein samples (40 μg) were separated by SDS‐PAGE and transferred to polyvinylidene difluoride (PVDF) membranes (Millipore, Billerica, MA). Then, the PVDF membranes were incubated with primary antibodies against PTEN, total AKT, phosphorylated AKT (p‐AKT) and GAPDH (Cell Signaling Technology, Danvers, MA, USA). Bands were visualized by detection with the ECL substrate.

### 2.6 Cell proliferation and cell cycle analyses

The EdU (5‐ethynyl‐2′‐deoxyuridine) cell proliferation was assessed using the Cell‐Light EdU Apollo567 In Vitro Imaging Kit (RiboBio, Guangzhou, China). Images were captured by fluorescence microscopy (Olympus Corp., Tokyo, Japan) and analysed in ImageJ (NIH, Bethesda, MD, USA). For a Cell Counting Kit 8 (CCK‐8) assay, cells were seeded in 96‐well plates at density 2000/well. After 24, 48, 72 and 96 hours, 10 μL of the CCK‐8 solution (Dojindo, Kumamoto, Japan) was added into each well, and the absorbance at 450 nm was measured after incubation for 2 hours. For cell cycle analysis, the cells were stained with propidium iodide (PI) via the Cycle TEST PLUS DNA Reagent Kit (BD Biosciences, Bedford, MD) and analysed by flow cytometry.

### 2.7 Cell migration and invasion assays

These assays were performed in a transwell chamber (8‐μm pore size, Corning, Cambridge, MA, USA). Cells were resuspended in 200 μL of the FBS‐free medium at a density of 2 × 10^5^ cells/mL and seeded in the upper chamber. Next, the cells were placed on the top side of the membrane (without Matrigel for the migration assay) or placed on the top side of the membrane precoated with Matrigel (BD Biosciences) (for the invasion assay). Forty‐eight hours later, the cells on the upper membrane migrated to or invaded the lower side of the membrane were then fixed in methanol and stained with 0.5% crystal violet. The average number of stained cells was determined and calculated in five randomly selected visual fields. Images were taken at the indicated time‐points (Olympus).

### 2.8 The luciferase reporter assay

The theoretical binding sequence for miR‐21 in the *LINC00312* gene and its mutant sequence were cloned into the psiCHECK‐2 vector (Promega, Madison, WI, USA) to construct a dual luciferase reporter plasmid. The wild‐type (wt) 3′‐UTR fragment of *PTEN* gene and its mutant (mut) of the miR‐21 binding site were cloned into a the psiCHECK‐2 vector to form the reporter vector PTEN‐3′‐UTR‐wt and PTEN‐3′‐UTR‐mut, respectively, as described previously.^15^ SW620 and LoVo cells were transfected with wt (or mut) reporter plasmid and an NC mimic or miR‐21 mimic for 48 hours. Luciferase activity was evaluated by means of a Luciferase Reporter Assay System (Promega). The *Renilla* luciferase/Firefly luciferase ratio was calculated to determine the differences between different alleles.

### 2.9 In vivo proliferation and metastasis assays

Animal experiments were approved by the Animal Care and Use Committee of Second Military Medical University (Shanghai, China) and were conducted following the animal treatment policies of Second Military Medical University in accordance with the National Institutes of Health guidelines. Five million SW620 cells overexpressing LINC00312 or control cells were subcutaneously injected into 5‐week‐old female BALB/c nude mice (n = 5 per group), and tumour growth was examined every 5 days for 30 days. The tumour volume was calculated according to the following formula: volume = length × width^2^ × 0.5. To assess the effect of LINC00312 on the metastatic ability of CRC in vivo, the established stably LINC00312‐overexpressing SW620 cells (5 × 10^6^) were injected into the spleen of nude mice. Six weeks later, the liver was excised and embedded in paraffin. Consecutive sections (4 μm thick) were prepared and stained with haematoxylin and eosin (H&E). H&E staining and morphological features were examined under a microscope to evaluate liver metastases.

### 2.10 Statistical analysis

Data are presented as mean ± standard deviation (SD) of at least three independent experiments. Statistical analysis was performed in SPSS 12.0 (SPSS, Inc. Chicago, IL, USA) and Origin 8.0 software. Differences between two groups or more than two groups were evaluated, respectively, by Student's *t* test or one‐way analysis of variance (ANOVA). The correlation between LINC00312 and miR‐21 expression levels was explored by Spearman's correlation method.

## 3 RESULTS

### 3.1 LINC00312 was found to be down‐regulated in CRC

**Figure 1:**
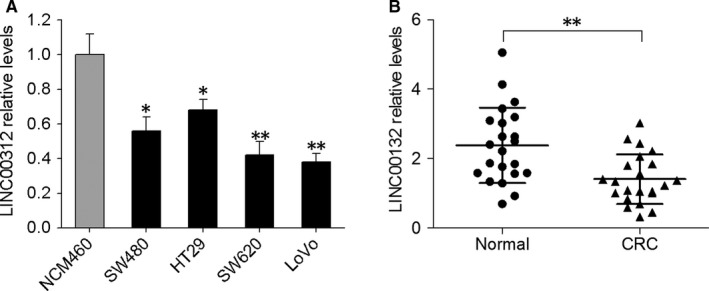
Expression of LINC00312 was low in both CRC tissues and cell lines. A, Expression of LINC00312 was detected by qRT‐PCR in normal colon epithelial cells and the four CRC cell lines. B, Expression of LINC00312 was compared between 22 CRC samples and the corresponding adjacent noncancerous tissues. GAPDH served as the endogenous control. Data are presented as mean ± SD; *P < 0.05, **P < 0.01

SYBR green qRT‐PCR was first carried out to determine LINC00312 levels in four human CRC cell lines (SW480, HT29, SW620, and LoVo) and in NCM460, the normal colon epithelial cell line. All four CRC cell lines showed notably reduced levels of LINC00312, whereas NCM460 cells expressed high levels of LINC00312 (Figure 1A). Furthermore, we detected LINC00312 in CRC tissues and adjacent noncancerous tissues from 22 patients. As shown in Figure 1B, LINC00312 expression was significantly lower in CRC tissues compared with the adjacent noncancerous tissues. These results supported the finding that LINC00312 is down‐regulated in CRC.

### 3.2 LINC00312 overexpression suppressed CRC cell proliferation, migration and invasion in vitro

**Figure 2:**
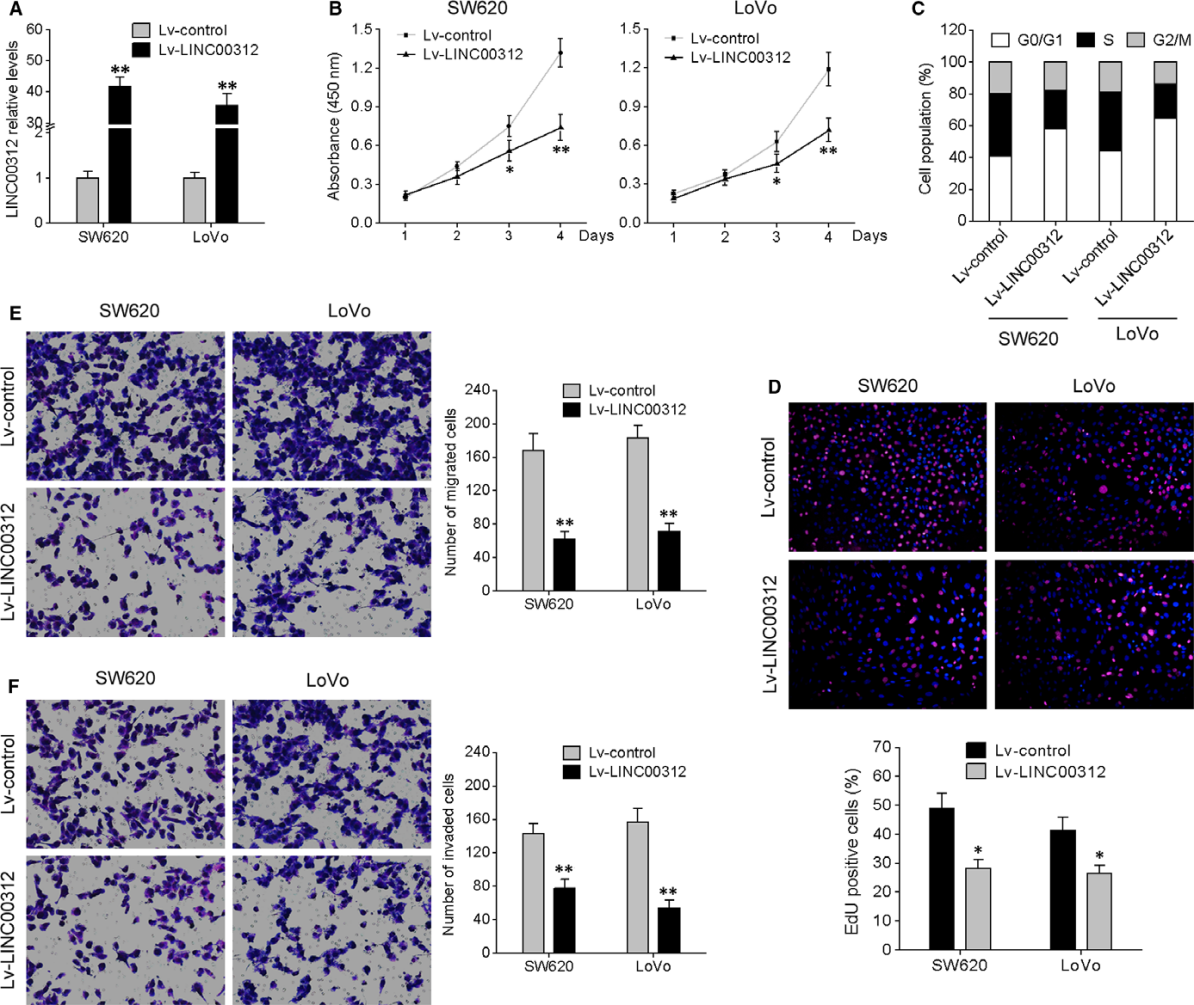
Overexpression of LINC00312 suppressed CRC cell proliferation, migration and invasion in vitro. A, SW620 and LoVo cells were infected with a LINC00312‐carrying lentivirus (Lv‐LINC00312) and a control lentivirus (Lv‐control), and qRT‐PCR was conducted to measure LINC00312 expression. B, Cell proliferation assay (CCK‐8). C, Flow‐cytometric analysis of the cell cycle distribution. D, Representative pictures (top) and quantification (bottom) of EdU‐positive cells. E, Transwell assay was performed to investigate changes in cell migration and invasiveness. Representative images (left) and quantification (right) are also shown. Data are presented as mean ± SD. *P < 0.05, **P < 0.01

To determine the function of LINC00312 in CRC, we first performed in vitro gain‐of‐function analyses by overexpressing LINC00312 via a lentiviral vector in SW620 and LoVo cells, which express LINC00312 relatively weakly. The successful increase in LINC00312 expression in these cells was confirmed by qRT‐PCR (Figure 2A). CCK‐8 analysis revealed that overexpression of LINC00312 significantly suppressed the growth of SW620 and LoVo cells when compared to their corresponding controls (Figure 2B). Flow cytometric analysis indicated that LINC00312 overexpression resulted in cell cycle arrest at the G0/G1 transition and a blockage in the S phase, indicating that DNA of S phase cells was damaged (Figure 2C). The EdU staining assay also revealed that the EdU incorporation drastically decreased after LINC00312 overexpression (Figure 2D). Subsequently, we determined whether LINC00312 can affect CRC cell migration and invasion. Transwell assays with or without Matrigel showed that LINC00312 overexpression suppressed CRC cell migration and invasion (Figure 2E,F). These data suggested that LINC00312 might act as a tumour suppressor in CRC.

### 3.3 LINC00312 directly interacts with miR‐21

**Figure 3:**
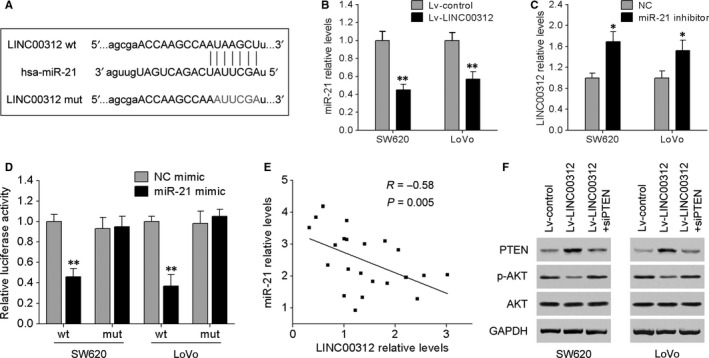
LINC00312 is a direct target of miR‐21. A, Bioinformatic prediction suggested that the LINC00312 sequence contains a putative binding site for miR‐21. B, miR‐21 expression decreased in SW620 and LoVo cells overexpressing LINC00312. C, LINC00312 expression increased in SW620 and LoVo cells transfected with the miR‐21 inhibitor, while this increase was counteracted by miR‐21 mimic transfection. D, A fragment of LINC00312 containing a wild‐type (wt) or mutated (mut) miR‐21‐binding site was inserted downstream of the luciferase gene within the reporter vector and cotransfected into SW620 and LoVo cells with the miR‐21 mimic or NC mimic. The relative luciferase activities are presented. E, Spearman's correlation analysis showed that miR‐21 expression levels inversely correlated with LINC00312 levels among CRC tissue samples (*R* = −0.58; *P* = 0.005). F, Western blotting analysis was carried out to detect the expression of PTEN, AKT and p‐AKTin SW620 and LoVo cells after transfection with LINC00312, si‐PTEN, or the empty vector. Data are presented as mean ± SD. *P < 0.05, **P < 0.01

To further uncover the molecular mechanism by which LINC00312 exerts its biological function in CRC, we performed bioinformatic analysis by different computational methods: miRanda and miRcode. Among the predicted miRNAs, we were particularly interested in miR‐21 because of its stimulatory effect on cancer cell proliferation and invasion.^16^ The predicted binding site of miR‐21 in the LINC00312 sequence is illustrated in Figure 3A. qRT‐PCR results indicated that overexpression of LINC00312 significantly reduced the miR‐21 expression in SW620 and LoVo cells (Figure 3B), and the expression levels of LINC00312 were up‐regulated by treatment with a miR‐21 inhibitor, while miR‐21 mimic transfection counteracted this up‐regulation (Figure 3C). We then constructed the luciferase reporter vectors containing a wild‐type (wt) or mutated (mut) miR‐21‐binding site in LINC00312 (Figure 3A). The results of dual‐luciferase reporter assays revealed that miR‐21 repressed the luciferase activity of the LINC00312‐wt reporter vector, but had no effect on the LINC00312‐mut vector (Figure 3D). Furthermore, there was a statistically significant inverse correlation between the levels of miR‐21 and LINC00312 among CRC tissue samples (Figure 3E). These data indicated that LINC00312 is a direct target of miR‐21.

### 3.4 The miR‐21–PTEN axis is involved the tumour‐suppressive effects of LINC00312

**Figure 4:**
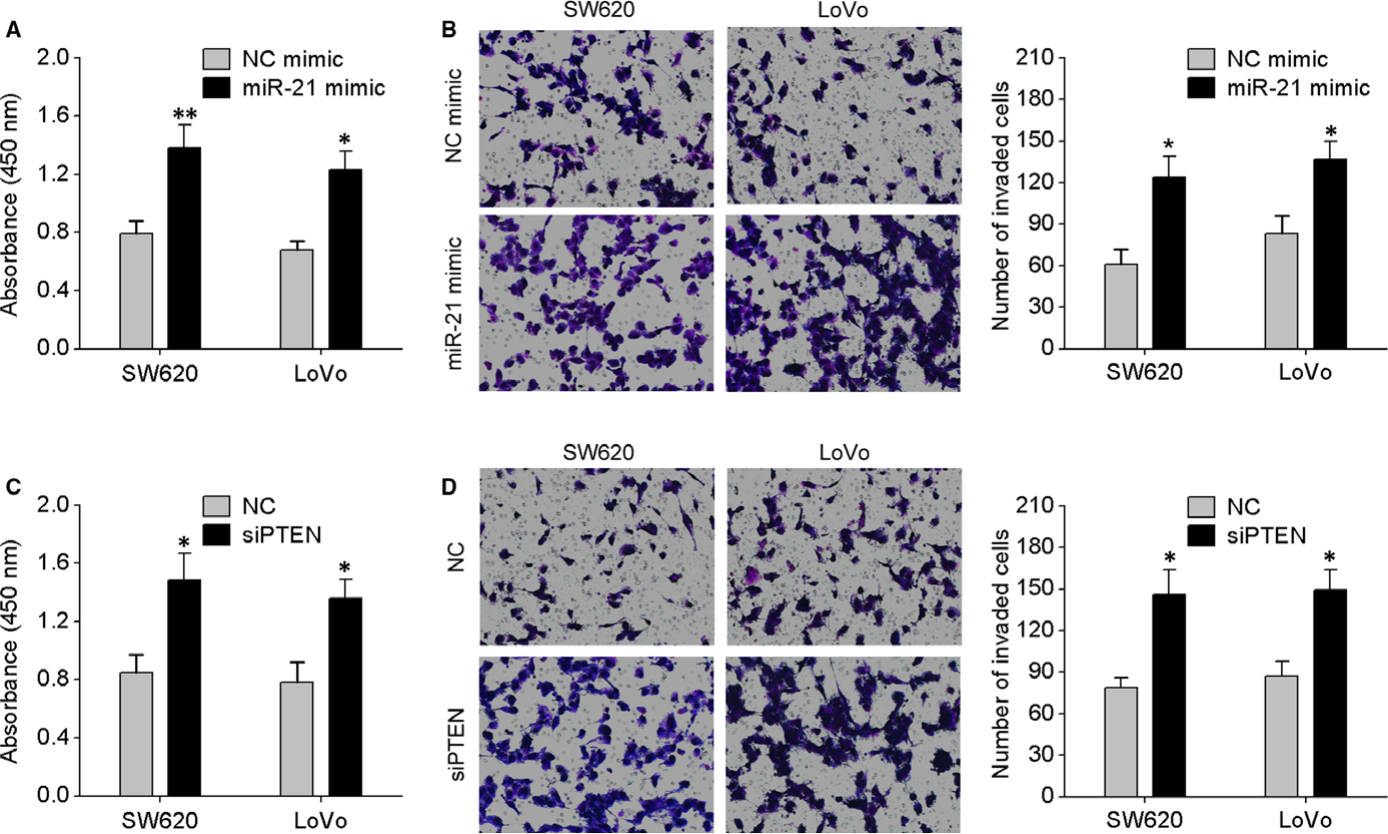
The miR‐21–PTEN axis is involved the antitumour effects of LINC00312 on CRC cells. A, LINC00312‐overexpressing SW620 and LoVo cells were transfected with miR‐21 mimic or NC mimic, and CCK‐8 assay was performed. B, Transwell invasion assay of the indicated cells. C, LINC00312‐overexpressing SW620 and LoVo cells were transfected with PTEN siRNA or NC, and the CCK‐8 assay was performed. D, The transwell invasion assay of the indicated cells. Data are presented as mean ± SD. *P < 0.05, **P < 0.01

After the finding that LINC00312 is a target of miR‐21, the role of miR‐21 in LINC00312‐driven inhibition of CRC progression was still unclear. We transfected miR‐21 mimic into LINC00312‐overexpressing SW620 and LoVo cells. The results showed that miR‐21 mimic strongly attenuated LINC00312‐induced inhibitory effects on CRC cell proliferation and invasion (Figure 4A,B), indicating that miR‐21 plays a crucial role in LINC00312‐induced antitumour effects on CRC cells.

It has been found that miR‐21 may promote CRC cell proliferation and invasion by down‐regulating PTEN protein expression.^15^, ^17^ Here, our results confirmed that miR‐21 significantly reduced PTEN levels in SW620 and LoVo cells by directly targeting its 3′UTR using luciferase reporter and Western blotting assays (Figure S1A,B). In addition, the expression levels of miR‐21 and PTEN were detected in four human CRC cell lines (SW480, HT29, SW620 and LoVo) and in NCM460. As indicated in Figure S1C,D, the cells with the relatively high endogenous miR‐21 expression level exhibited low levels of PTEN. On the basis of these data, we speculated that PTEN is involved in the LINC00312/miR‐21‐dependent malignant progression of CRC. We cotransfected SW620 and LoVo cells with LINC00312 and PTEN siRNA as described in Figure 3F, and the expression of PTEN was confirmed by Western blotting. Functional assays showed that knockdown of PTEN with siRNA dramatically reversed the suppressive effects of LINC00312 on CRC cell proliferation and invasion (Figure 4C,D). Given that AKT was an important downstream target of PTEN, the levels of p‐AKT and AKT were also examined. Figure 3F shows that PTEN knockdown abrogated the decreased expression of p‐AKT induced by LINC00312 in CRC cells. These results suggested that LINC00312 acted as a tumour suppressor via inhibition of miR‐21 and by targeting PTEN.

### 3.5 LINC00312 inhibits CRC tumour growth and liver metastases in vivo

**Figure 5:**
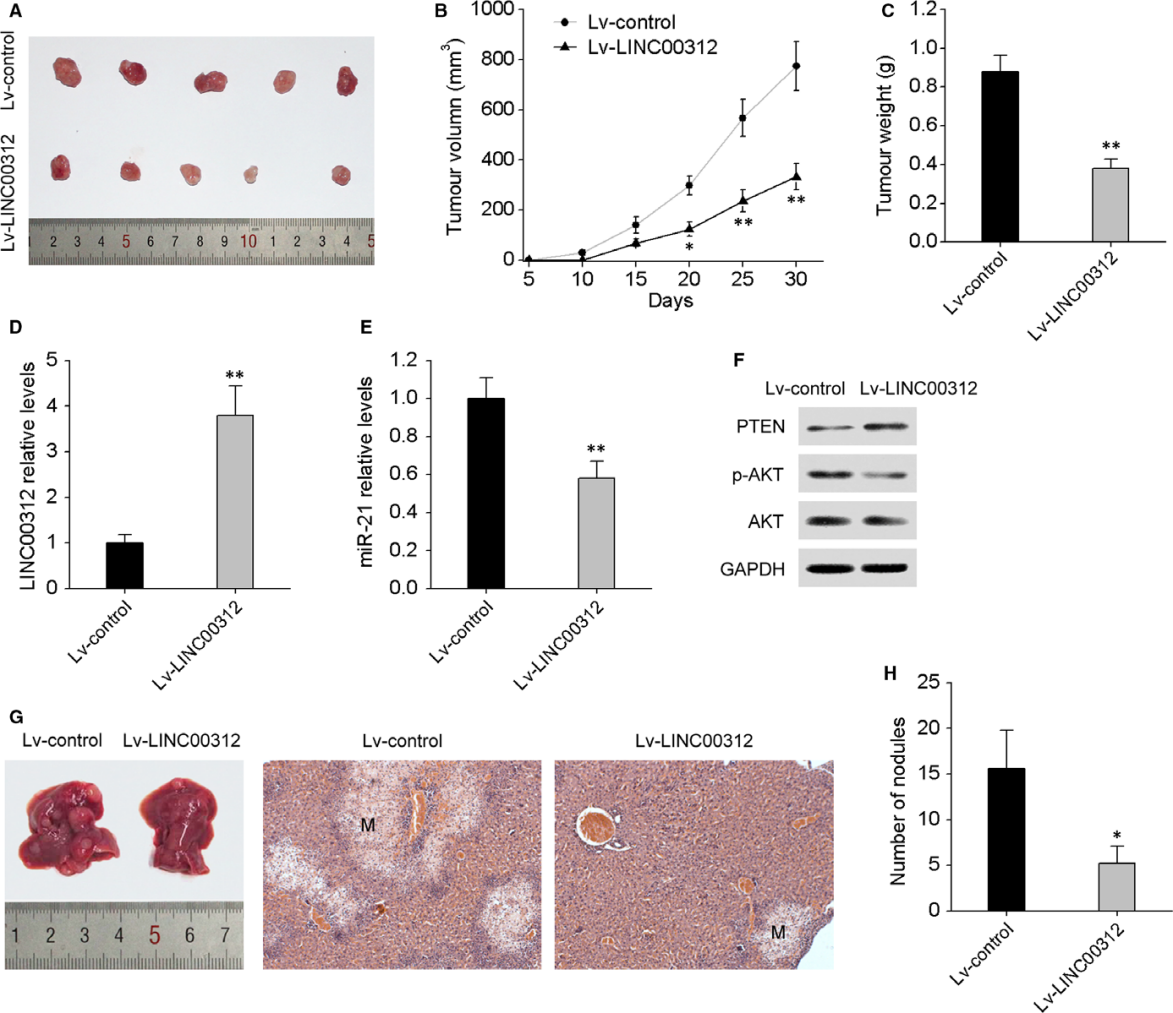
LINC00312 suppressed tumour growth and metastasis in nude mice. A, A representative photograph of tumour formation: LINC00312‐overexpressing SW620 cells were subcutaneously injected into nude mice; data were collected for up to 30 days. The growth curves of tumour volumes (B) and weight (C) are depicted. Levels of LINC00312 (D) and miR‐21 (E) in xenograft tumour tissues as determined by qRT‐PCR. F, Western blot analyses of PTEN, p‐AKT and AKT in LINC00312 overexpressing and control xenograft tumour tissues. G, LINC00312‐overexpressing SW620 cells were injected into nude mice through the spleen to evaluate the liver metastatic potential of the cells. After 6 weeks, the livers were sectioned for examination of tumour metastasis by H&E staining. H, The number of metastatic liver nodules per mouse. Data are presented as mean ± SD. *P < 0.05, **P < 0.01

To test whether LINC00312 expression can affect tumour growth in vivo, SW620 cells overexpressing LINC00312 were inoculated into nude mice. At 30 days after the injection, the tumours in the LINC00312 overexpression group were smaller than those in the control group (Figure 5A). The mean tumour volume and weight in the LINC00312 overexpression group were significantly lower than those in the control group (Figure 5B,C). qRT‐PCR and Western blotting analyses of the tumour tissues confirmed elevated LINC00312 with decreased miR‐21 and p‐AKT, and increased PTEN expression in LINC00312 overexpressing tumours (Figure 5D‐F). Furthermore, we examined the influence of LINC00312 overexpression on a murine model of CRC metastasis. We implanted LINC00312‐overexpressing SW620 cells or control cells into nude mice through spleen injection. Six weeks after the transplant, the mice were anaesthetized, and the livers were dissected for macroscopic and microscopic histological analyses. Livers from mice that received a transplant of LINC00312‐overexpressing SW620 cells formed more colonization foci than did control cells (Figure 5G,H). Taken together, these results indicated that overexpression of LINC00312 inhibited tumour growth and metastasis in nude mice.

## 4 DISCUSSION

In this study, we demonstrated that LINC00312 is down‐regulated in human CRC tissues and cell lines. Overexpression of LINC00312 suppressed in vitro cell proliferation and invasion and restrained in vivo tumour growth and metastasis. We also validated the antitumour function of LINC00312 by modulating the miR‐21–PTEN pathway during CRC development. These results suggest that LINC00312 may serve as a tumour suppressor in CRC and imply a new therapeutic strategy against such malignant tumours.

Recent studies showed that LINC00312 is associated with several cancers.^11^, ^18^, ^19^ Zhang et al. found that LINC00312 inhibits proliferation and invasiveness of nasopharyngeal carcinoma cells.^11^ Wang et al. and Liu et al. revealed that overexpression of LINC00312 inhibits the invasion and migration of bladder and thyroid cancer cells.^18^, ^20^ Zhu et al. reported that LINC00312 inhibits cell proliferation and promotes apoptosis via HOXA5 in non‐small cell lung cancer.^19^ A recent study has shown that LINC00312 is down‐regulated in CRC tissues with metastasis compared with CRC tissues without metastasis by miRNA microarray analysis,^21^ suggesting that LINC00312 may be involved in CRC progression. Here, our results show that LINC00312 is significantly down‐regulated in CRC tissues and cell lines. In vitro and in vivo assays indicated that overexpression of LINC00312 significantly inhibits CRC cell growth and metastasis.

One of the interesting cross‐regulatory mechanisms between lncRNAs and miRNAs is the function of a competing endogenous RNA or a molecular sponge.^22^ For instance, Wang et al. have found that DLEU1 contributes to ovarian carcinoma tumourigenesis and progression by interacting with miR‐490‐3p and altering CDK1 expression. Cui et al. have reported that lncRNA CCAT1 promotes glioma tumourigenesis by sponging miR‐181b, thereby leading to derepression of its endogenous targets FGFR3 and PDGFRα; these findings point to a potential therapeutic target in glioma.^23^, ^24^ A recent study verified that LINC00312 is associated with miR‐197‐3p and modulates the expression of a miR‐197‐3p target, p120, thereby providing powerful evidence that LINC00312 enhances gene expression at the post‐transcriptional level.^20^ Here, we revealed a potential binding site within LINC00312 and miR‐21 by bioinformatic prediction. Given that miR‐21 is overexpressed in CRC^25^, ^26^, ^27^ and promotes cell growth and invasion by repressing PTEN in CRC,^15^, ^17^ we hypothesized that LINC00312 might function as a miR‐21 sponge to enhance PTEN expression. Our luciferase reporter assay showed that miR‐21 is a direct interaction partner of LINC00312 in CRC cells. LINC00312 significantly suppressed miR‐21 expression, enhanced PTEN expression and decreased p‐AKT level. Furthermore, miR‐21 mimics attenuated the inhibitory effects of LINC00312 on CRC cell proliferation, migration and invasion; these phenomena were similar to the effects of PTEN siRNA on LINC00312‐overexpressing cells. These findings suggest that the miR‐21–PTEN pathway is involved in the LINC00312‐controlled malignant progression of CRC.

Taken together, our data imply that LINC00312 serves as a tumour suppressor inhibiting CRC growth and metastasis in vitro and in vivo. Moreover, we found that LINC00312 promotes the expression of PTEN by sponging miR‐21 during CRC progression and therefore may be a novel therapeutic target in CRC.

## CONFLICT OF INTEREST

The authors declare no conflict of interest.
