# Supplementary figures and images for: LINC00312 represses proliferation and metastasis of colorectal cancer cells by regulation of miR‐21
Source: J Cell Mol Med. 2018 Aug 22;22(11):5565–72. doi: 10.1111/jcmm.13830 (PMC6201213; doi:10.1111/jcmm.13830)

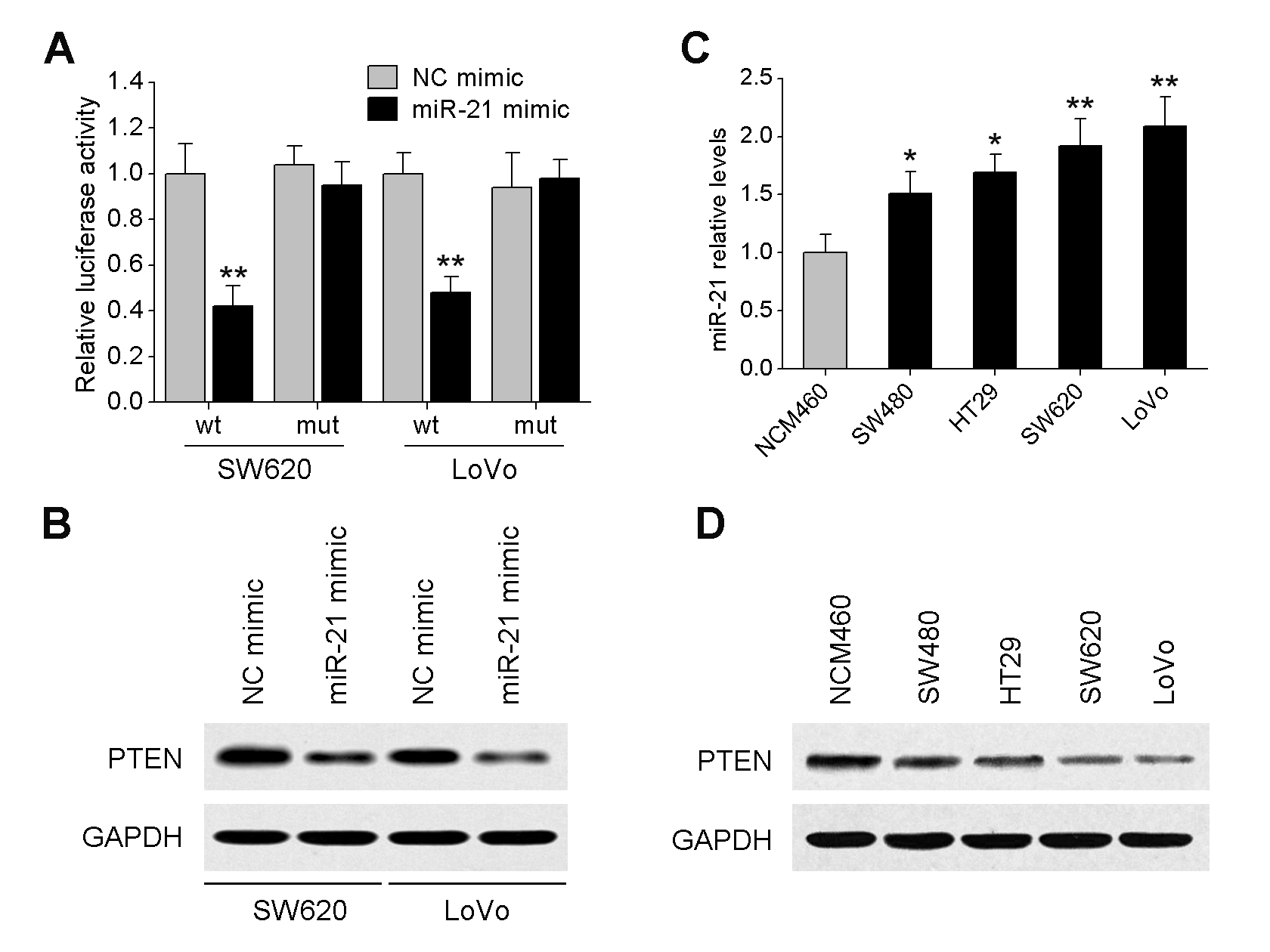

Supplement: Supplementary file 1 [file JCMM-22-5565-s001.tif]
