# Supplementary material for: LINC00312 represses proliferation and metastasis of colorectal cancer cells by regulation of miR‐21
Source: J Cell Mol Med. 2018 Aug 22;22(11):5565–72. doi: 10.1111/jcmm.13830 (PMC6201213; doi:10.1111/jcmm.13830)
Supplement: Supplementary file 2 [file JCMM-22-5565-s002.docx]

**Supplemental figures**


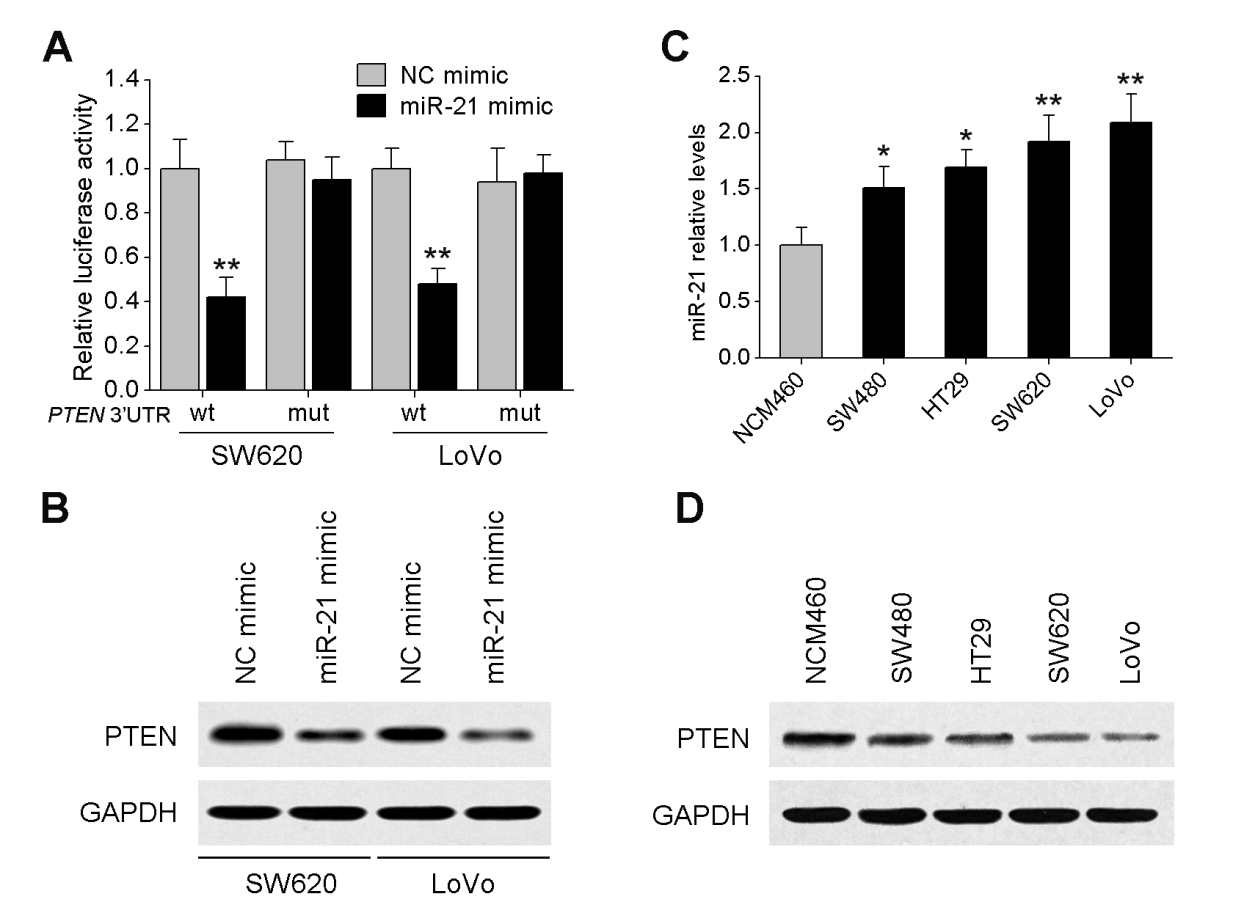


**Supplementary figure 1.** miR-21 downregulates PTEN by targeting its 3'UTR. (A) Relative luciferase activity in SW620 and LoVo cells cotransfected with wild-type (wt) or mutant (mut) PTEN-3'UTR plus miR-21 mimic or NC mimic. (B) Western blotting analysis of the expression of PTEN in SW620 and LoVo cells transfected with miR-21 mimic or NC mimic. (C) qRT-PCR analysis of the expression of miR-21 in four human CRC cell lines (SW480, HT29, SW620, and LoVo) and in the normal colon epithelial cell line NCM460. (D) Western blotting analysis of the expression of PTEN in the indicated cells. Data are presented as mean ± SD. *P < 0.05, **P < 0.01.
